# Genetic causal association between frozen shoulder and carpal tunnel syndrome: a two-sample mendelian randomization

**DOI:** 10.1186/s12891-024-07186-7

**Published:** 2024-01-13

**Authors:** Yang Chen, Xiaojin Wu, Yongxing Zhang, Jian Chen

**Affiliations:** 1 Department of Ultrasound, Sir Run Run Shaw Hospital, Zhejiang University School of Medicine, Hangzhou, Zhejiang China; 2 Department of Ultrasound, The Fourth Affiliated Hospital, Zhejiang University School of Medicine, Yiwu, Zhejiang China; 3 Department of Orthopedics, The Second Affiliated Hospital, Zhejiang University School of Medicine, Hangzhou, Zhejiang China

**Keywords:** Frozen shoulder (FS), Carpal tunnel syndrome (CTS), Mendelian randomization (MR), Genome-wide association study (GWAS), Genetic causality, Single nucleotide polymorphism (SNP)

## Abstract

**Objective:**

Observational studies have suggested an association between frozen shoulder (FS) and carpal tunnel syndrome (CTS). However, due to challenges in establishing a temporal sequence, the causal relationship between these two conditions remains elusive. This study, based on aggregated data from large-scale population-wide genome-wide association studies (GWAS), investigates the genetic causality between FS and CTS.

**Methods:**

Initially, a series of quality control measures were employed to select single nucleotide polymorphisms (SNPs) closely associated with the exposure factors. Two-sample Mendelian randomization (MR) was utilized to examine the genetic causality between FS and CTS, employing methods including Inverse-Variance Weighted (IVW), MR-Egger, Weighted Median, Simple Mode, and Weighted Mode approaches. Subsequently, sensitivity analyses were conducted to assess the robustness of the MR analysis results.

**Results:**

IVW analysis results indicate a positive causal relationship between CTS and FS (*p* < 0.05, OR > 1), while a negative causal relationship between the two conditions was not observed. Heterogeneity tests suggest minimal heterogeneity in our IVW analysis results (*p* > 0.05). Multivariable MR testing also indicates no pleiotropy in our IVW analysis (*p* > 0.05), and stepwise exclusion tests demonstrate the reliability and stability of the MR analysis results. Gene Ontology (GO) pathway analysis reveals enrichment of genes regulated by the associated SNPs in the TGFβ-related pathways.

**Conclusion:**

This study provides evidence of the genetic causal association between frozen shoulder and carpal tunnel syndrome and provides new insights into the genetics of fibrotic disorders.

**Supplementary Information:**

The online version contains supplementary material available at 10.1186/s12891-024-07186-7.

## Introduction

Adhesive Capsulitis, commonly known as frozen shoulder (FS), is a prevalent degenerative condition. The incidence of FS is 8% in males and 10% in females, with an average prevalence of approximately 2–5% [1, 2]. It typically manifests between the ages of 40 to 60 years [3]. FS is characterized by a gradual reduction in shoulder joint mobility accompanied by shoulder pain. It may develop after prolonged immobilization or be associated with systemic illnesses, often exhibiting an extended clinical course [4]. Patients often experience three overlapping phases: the inflammatory phase (pain and decreased range of motion), the frozen phase (stiffness), and the thawing phase (symptom resolution) [5]. The pathological hallmark of FS is fibroproliferative tissue fibrosis, characterized by the transformation of myofibroblasts from fibroblasts that predominantly produce Type I and Type III collagen, accompanied by inflammation, neovascularization and new nerve innervation. These processes ultimately result in fibrotic contraction and stiffness of the shoulder joint capsule [6]. The exact etiology of FS remains unclear, underscoring the imperative need for proactive exploration to inform treatment strategies and interventions for this condition.

Carpal tunnel syndrome (CTS) is the most prevalent surgically treated problem in the hand with the prevalence of 1–3% [7]. CTS primarily presents with symptoms such as pain, sensory abnormalities, and numbness in the hand, resulting from compression of tendons and the median nerve within the carpal tunnel. The pathophysiology of CTS remains poorly understood, with tendons and the median nerve within the carpal tunnel encapsulated by multiple layers of surrounding tissue known as subsynovial connective tissue (SSCT). Studies indicate that histological examinations of SSCT in CTS patients most commonly reveal non-inflammatory fibrosis within the carpal tunnel, leading to increased pressure within the carpal tunnel and nerve dysfunction [8]. Furthermore, the diminished capacity of fibrotic SSCT to withstand any new mechanical loads increases the likelihood of recurring fibrosis, thus perpetuating a vicious cycle [9].

Research indicates that there are certain commonalities in the pathogenesis of CTS and FS. However, further investigation is needed to elucidate the relationship between these two conditions and their respective mechanisms of onset. In epidemiological studies, Mendelian randomization (MR) is increasingly employed for inferring genetic causal relationships [10]. MR utilizes genetic markers closely associated with specific potential exposure factors as “instrumental variables” to assess the relationship between exposure factors and outcomes. Individual variations in genetic alleles, known as single nucleotide polymorphisms (SNPs), are exploited in MR because genetic markers (or alleles) are randomly allocated at conception, thereby leveraging this natural genetic variation to mitigate issues of confounding and reverse causation [11]. To date, there is a lack of research on the causal relationship between CTS and FS. Therefore, this study aims to investigate the causal relationship and underlying mechanisms of CTS and FS through a two-sample Mendelian randomization approach, utilizing the IEU OpenGWAS database. The goal is to provide novel strategies and treatment approaches for the diagnosis and management of these two diseases.

## Materials and methods

### Data search and filtering

The study was conducted using a two-sample Mendelian randomization (MR) approach. Firstly, we conducted a search in the IEU OpenGWAS database (https://gwas.mrcieu.ac.uk/) using the keyword “Carpal tunnel syndrome” to obtain the “ukb-d-G6_CARPTU” dataset, which was utilized as the “exposure factor.” Subsequently, we performed a search using the keyword “Frozen shoulder” to obtain the “ebi-a-GCST90000512” dataset, which was used as the “outcome " (forward MR analysis). Following that, we conducted an analysis in the reverse direction (reverse MR analysis) using the “ukb-d-G6_CARPTU” dataset as the “outcome " and the “ebi-a-GCST90000512” dataset as the “exposure factor”, enabling bidirectional investigation of the relationship between the exposure factor and outcome. The initial GWAS had obtained approval from the relevant institutional review board, and a detailed description of the research process can be found in our previously published study [12].

### Data pre-processing

The exposure factor was retrieved and instrumental variables were selected using the “extract_instruments” function in the R package “TwoSampleMR”. The selection criteria included: *p*-value threshold of 5*10^− 7^, identifying SNPs significantly associated with the exposure factor; clump = TRUE, removing linkage disequilibrium from instrumental variables; r^2^ = 0.001; and kb = 10,000.

The outcome variable data was obtained using the “extract_outcome_data” function in the R package “TwoSampleMR”. Relevant SNPs associated with the outcome were extracted and filtered in conjunction with instrumental variables from the exposure factor. The filtering criteria included: proxies = TRUE; rsq = 0.8, excluding SNPs with intermediate allele frequencies.

### Mendelian randomization (MR) analysis

We employed the “harmonise_data” function within the R package “TwoSampleMR” to standardize effect alleles and effect sizes. Subsequently, we conducted Mendelian randomization (MR) analysis using the “mr” function, incorporating five algorithms (MR Egger, Weighted median, Inverse variance weighted, Simple mode, Weighted mode). To assess the randomness of our analysis, for example, whether MR conforms to Mendel’s Second Law of random assortment, we created funnel plots by combining the β and standard error (SE) of each instrumental variable.

### Sensitivity analysis

To evaluate the reliability of the MR analysis results, we conducted sensitivity analysis, including heterogeneity testing (using the “mr_heterogeneity” function in the R package “TwoSampleMR”), horizontal pleiotropy testing (using the “mr_pleiotropy_test” function in the R package “TwoSampleMR”), and leave-one-out analysis (employing the “mr_leaveoneout” function in the R package “TwoSampleMR”).

### Expression quantitative trait loci (eQTL) analysis

To explore the role of instrumental variables in causal mediation between the exposure factor and outcome, we leveraged the instrumental variable SNPs obtained from MR analysis. Combining these SNPs with data from the eQTLGen database, we investigated genes that regulate gene expression from the perspective of cis-eQTL (cis-expression quantitative trait loci). Gene ontology (GO) enrichment analysis of the involved genes was performed using the R package “clusterProfiler” to explore gene expression regulation patterns.

## Results

Details of the datasets used in this study are provided in Table S1 (see Supplementary materials).

### Characteristics of instrumental variables

After rigorous selection, we identified 13 SNPs associated with CTS (exposure factor). Combining these with the outcome (FS), we further filtered out SNPs with intermediate allele frequencies, leaving us with a set of 12 instrumental variables (SNPs) related to the outcome (Table S2). Applying the same approach, we reversed the exposure and outcome variables, treating FS as the exposure factor and CTS as the outcome, ultimately yielding 8 instrumental variables (SNPs) related to the outcome (Table S2).

### Genetic causal relationship between CTS and FS

**Fig. 1:**
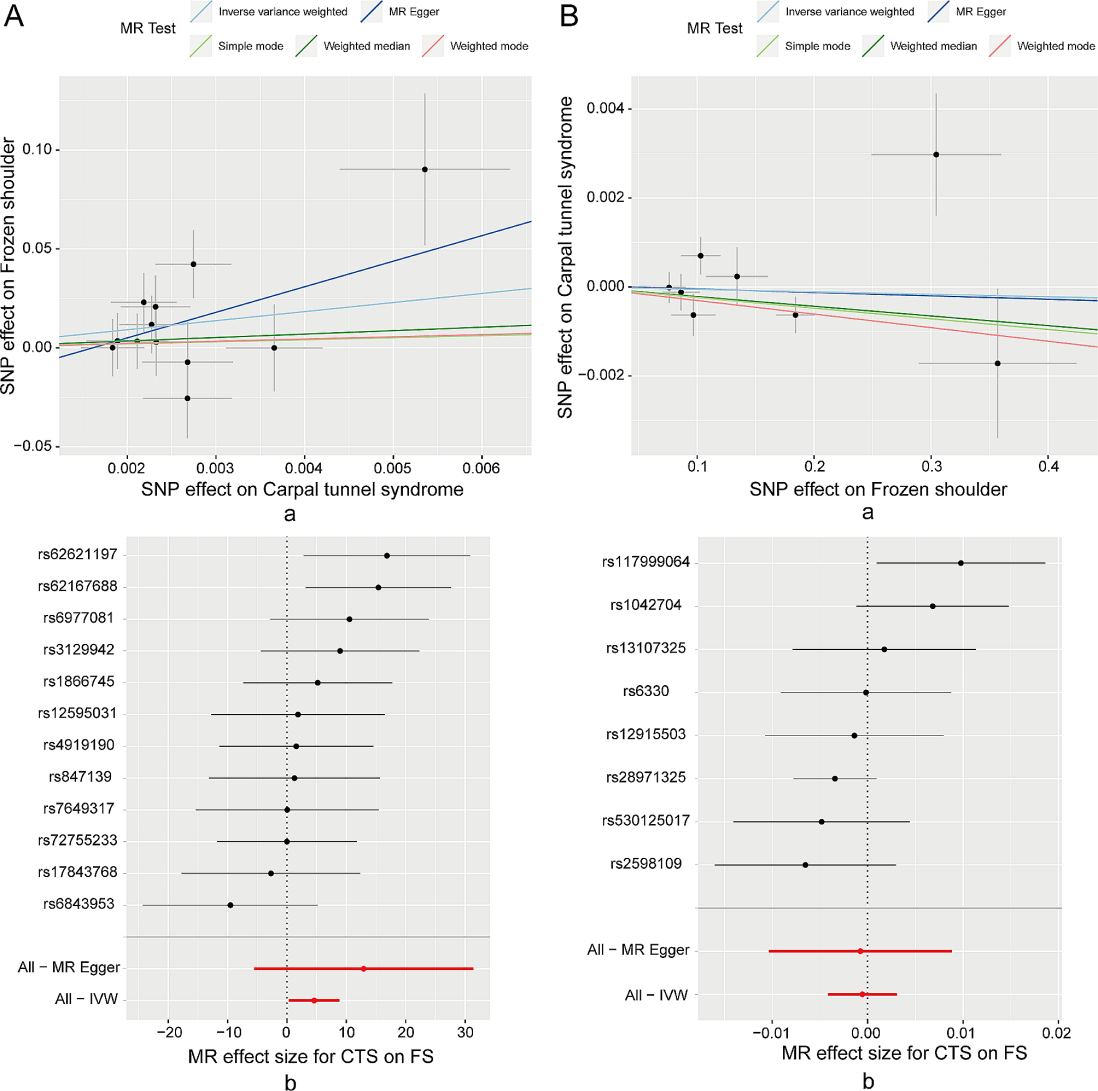
The overall effect of the instrumental variable SNPs on both CTS and FS. **A)** Scatterplot **(a)** and forestplot **(b)** of SNP effects on CTS as exposure and FS as outcome; **B)** Scatterplot **(a)** and forestplot **(b)** of SNP effects on FS as exposure and CTS as outcome

In the forward Mendelian randomization (MR) analysis, the Inverse Variance Weighted algorithm exhibited statistical significance (*p* < 0.05) with an OR > 1 (Table 1). The overall effect of the SNPs on both CTS and FS demonstrated a positive correlation trend, with effect sizes consistently greater than zero (Fig. 1A), implying that CTS is a risk factor for FS. Conversely, in the reverse MR analysis, none of the five algorithms showed statistical significance (*p* > 0.05, Table 1). Scatterplots and forest plots revealed an indistinct overall trend (Fig. 1B), suggesting that FS does not significantly impact CTS.

**Table 1:** Mendelian randomization (MR) analysis of FS and CTS

Exposure	CTS			FS		
Outcome	FS			CTS		
Method	SNP (n)	OR (95%CI)	*P* value	SNP (n)	OR (95%CI)	*P* value
**MR Egger**	12	4.1E + 05 (4.0E-03 ~ 4.2E + 13)	0.20	8	0.9992 (0.9897–1.009)	0.8809
**Weighted median**	12	5.7 (2.5E-03 ~ 1.3E + 03)	0.54	8	0.9978 (0.9941–1.002)	0.2543
**Inverse variance weighted**	12	9.8E + 01 (1.4E + 00 ~ 7.0E + 03)	0.035*	8	0.9994 (0.9958–1.003)	0.7655
**Simple mode**	12	2.8 (1.9E-04 ~ 4.1E + 04)	0.84	8	0.9976 (0.9909–1.004)	0.5076
**Weighted mode**	12	3.1 (3.0E-04 ~ 3.1E + 04)	0.82	8	0.9970 (0.9930–1.001)	0.1769

Note: * *p* < 0.05

### The randomness of the MR analysis

**Fig. 2:**
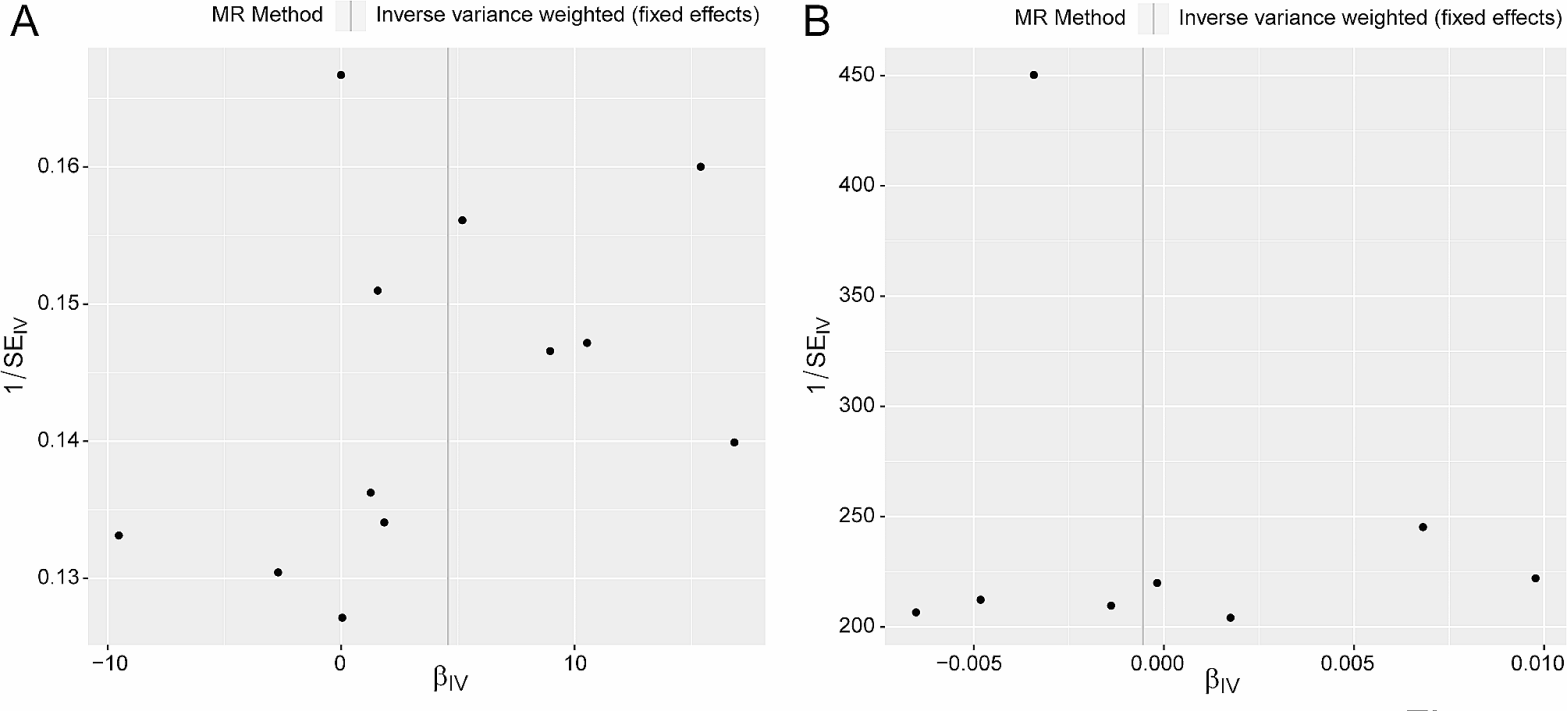
Funnel plots constructed by combining the β and standard error (SE) of each instrumental variable in the forward MR analysis (CTS as exposure and FS as outcome, **A**) and the reverse MR analysis (FS as exposure and CTS as outcome, **B**)

To determine if the analysis adhered to Mendel’s Second Law of random assortment. Funnel plots were constructed by combining the β and standard error (SE) of each instrumental variable (Fig. 2). In the forward MR analysis (Fig. 2A), instrumental variables were distributed symmetrically on both sides of the Inverse Variance Weighted (IVW) line, suggesting that the forward MR analysis conforms to randomness. Similarly, in the reverse MR analysis (Fig. 2B), instrumental variables exhibited relative symmetry on both sides of the IVW line, indicating that the reverse MR analysis also adheres to randomness.

### The sensitivity analysis of the MR study

**Fig. 3:**
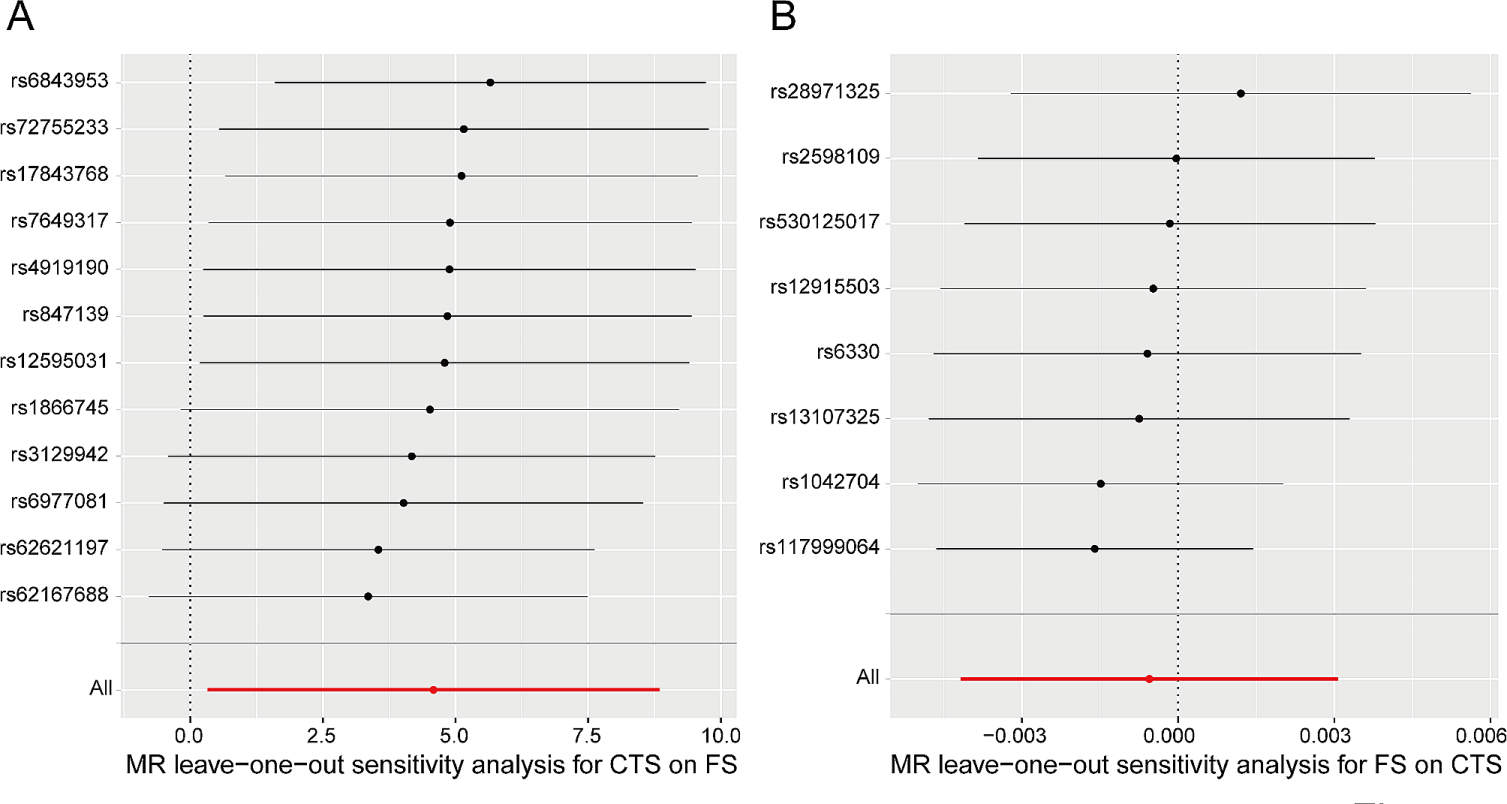
Leave-one-out analysis employing the inverse variance weighted (IVW) method in the forward MR analysis (CTS as exposure and FS as outcome, **A**) and the reverse MR analysis (FS as exposure and CTS as outcome, **B**)

1. **Heterogeneity analysis**: In this MR analysis, as we conducted a two-sample approach using SNP-exposure and SNP-outcome data, potential differences in populations and sequencing methodologies could introduce heterogeneity among the samples, which might impact the analysis results. Therefore, to assess the influence of heterogeneity, we conducted a heterogeneity analysis. The results indicated that all *P*-values were greater than 0.05 (Table 2), signifying the absence of heterogeneity between the two sets of samples.
2. **Horizontal pleiotropy test**: to evaluate the presence of confounding factors in this study, we performed a horizontal pleiotropy test (Table 2). The results showed that the *P*-value was also greater than 0.05, indicating the absence of confounding factors in the study, further ensuring the reliability of the analysis results.
3. **Leave-one-out analysis**: to assess whether individual instrumental variables significantly altered the outcome, we conducted a leave-one-out analysis. The purpose was to systematically remove each SNP, calculate the meta-effect of the remaining SNPs, and observe if the results changed, and this analysis employed the inverse variance weighted method (Fig. 3). The results demonstrated that the effect of the remaining SNPs on the outcome did not change significantly with the stepwise removal of each SNP, indicating the reliability and stability of the MR analysis results.

**Table 2:** Sensitivity analysis of the MR analysis results of FS and CTS

	outcome	exposure	method	*P* value
**Heterogeneity**	FS	CTS	Inverse variance weighted	0.2923
	CTS	FS		0.07745
**pleiotropy**	FS	CTS	Egger intercept	0.3839
	CTS	FS		0.9635

### SNP and SNP-regulating gene pattern analysis

**Fig. 4:**
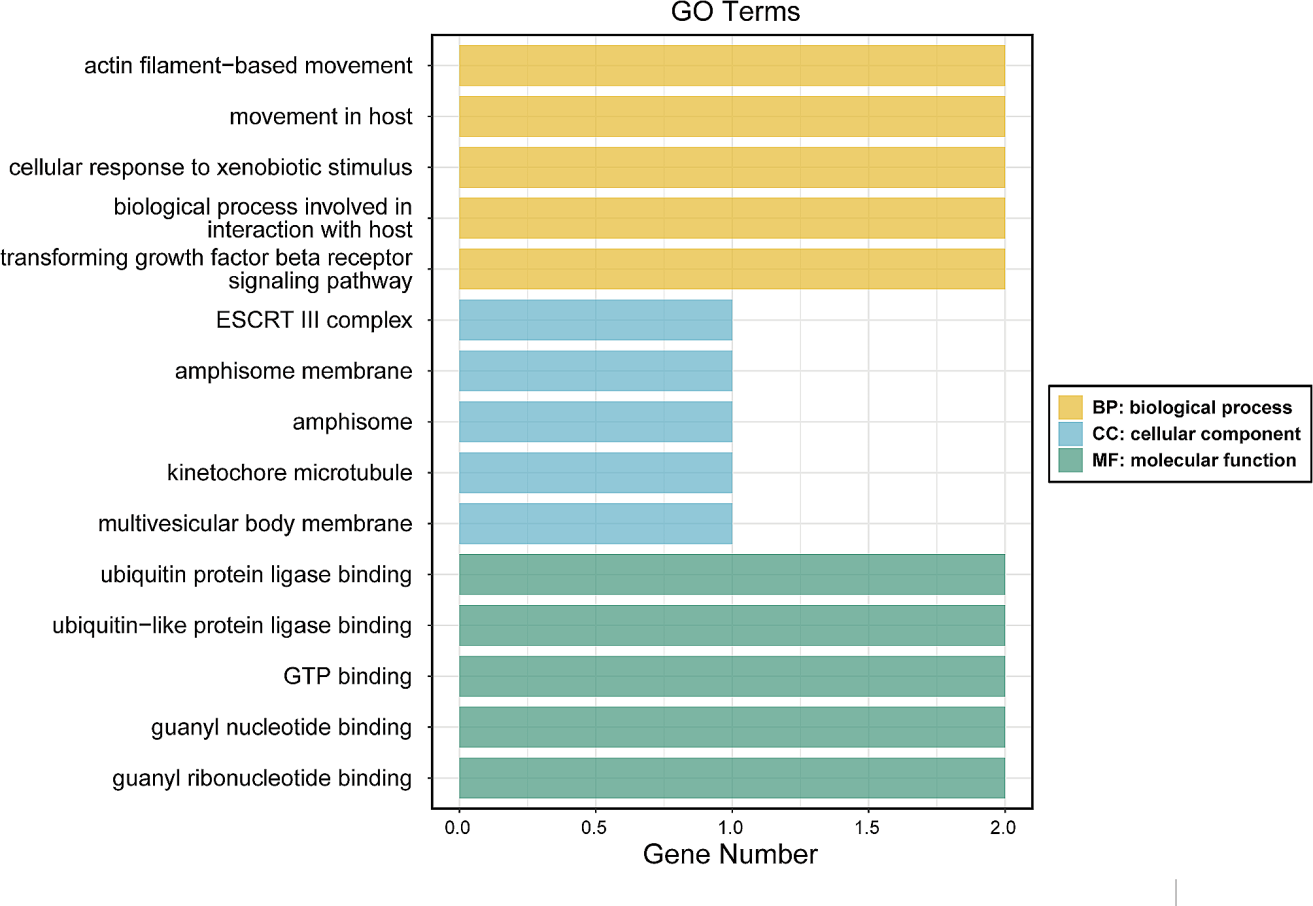
The GO enrichment analysis of the 14 genes from a cis-eQTL perspective of 12 SNPs in the forward MR analysis (CTS as exposure and FS as outcome)

To explore the role of instrumental variables in the causal mediation between the exposure factor and the outcome, we leveraged instrumental variable SNPs obtained through MR analysis and combined them with data from the eQTLGen database to investigate genes that regulate gene expression from a cis-expression quantitative trait locus (cis-eQTL) perspective. In the set of instrumental variables from the forward MR analysis, a total of 14 genes were identified from 12 SNPs: R3HCC1L, UMPS, KIAA1715, LOXL4, CHMP2B, ITGB5, MYO1F, AGAP3, PYROXD2, SMEK2, GIMAP8, KCNH2, SMAD6, and CTD-2054N24.2, exhibiting cis-regulatory control over gene expression. The GO enrichment analysis of these 14 genes yielded 211 significant results (*p* < 0.05), encompassing 163 biological processes, 16 cellular components, and 32 molecular functions related to various pathways (Fig. 4). Notably, ITGB5 and SMAD6 were enriched in the transforming growth factor beta (TGFβ) receptor signaling pathway, indicating potential involvement of the TGFβ/SMAD (Suppressor of Mothers against Decapentaplegic, a well-conserved family of transcriptional factors) regulatory pathway in the development of CTS and FS.

## Discussion

In this study, we conducted a bidirectional Mendelian randomization analysis combined with GWAS datasets to examine and explore the relationship between carpal tunnel syndrome (CTS) and frozen shoulder (FS) from a genetic perspective. Firstly, we identified 12 instrumental variables (SNPs) associated with CTS, which effectively predicted an increased risk of FS. However, the impact of FS on CTS was not significant. Next, we subjected the results of both forward and reverse Mendelian randomization analyses to sensitivity analyses, which included tests for heterogeneity, horizontal pleiotropy, and leave-one-out assessments. The findings revealed the following: (1) There was no heterogeneity between the two-sample datasets chosen for this analysis. (2) Exposure factors did not exhibit additional confounding factors when influencing the outcome through instrumental variables; (3) Overall, the instrumental variables showed stable effect estimates on the outcome. These three aspects collectively ensure the reliability of the MR analysis results. Last but not least, we conducted a Gene Ontology (GO) analysis for the 12 SNPs associated with CTS and increasing the risk of FS, along with the 14 genes they regulate. The analysis revealed enrichment in the TGFβ receptor signaling pathway, suggesting potential involvement of the TGFβ/SMAD regulatory pathway in the development of both CTS and FS.

From a pathogenic perspective, both FS and CTS are closely associated with connective tissue fibrosis. Research has indicated that specific differential finger movements may cause damage to the subsynovial connective tissue (SSCT) and lead to fibrosis due to traumatic injury [13]. Injured SSCT thickens and accumulates interstitial fluid, resulting in a narrowing of the carpal tunnel space, which in turn restricts the movement of tendons and the median nerve. In the case of FS, pain precedes stiffness in the course of the disease. This suggests an evolution from inflammation to fibrosis in the shoulder joint capsule during the development of FS [14]. Arthroscopic findings have revealed pathological features similar to palmar fascia contracture syndrome, characterized by connective tissue fibrosis around the biceps tendon and surrounding muscle tissues [4, 15]. Histologically, it is indicated that myofibroblasts replace fibroblasts, and an excess secretion of type III collagen fibers densely arranged around the joint capsule leads to restricted movement [16]. However, these findings are observed in patients with severe late-stage FS and may not be applicable to early stages. The differences in the fibrotic stage over the course of the disease may explain why CTS can serve as a risk predictor for FS, but not vice versa. Simultaneously, recognizing CTS as a manifestation of a systemic disorder may contribute to the early intervention in other fibrotic disorders such as adhesive capsulitis in knee and hip and elbow stiffness. This suggests that uncovering the genetic causal relationship between CTS and FS holds significant clinical implications for the diagnosis and treatment of fibrotic lesions in peripheral joints.

Research has demonstrated that TGFβ can promote fibrosis through three mechanisms [17, 18]: (1) TGFβ1 inhibits extracellular matrix (ECM) degradation by suppressing matrix metallopeptidase (MMP) expression or promoting its inhibitor TIMP expression; (2) TGFβ1 induces myofibroblast formation through the EMT effect; (3) TGFβ1 induces ECM production through SMAD3-dependent or non-SMAD-related mechanisms. The transcription factor SMAD proteins, as key intracellular effectors of TGFβ1, have been extensively studied [19]. Generally, SMAD3 and SMAD4 promote fibrosis, while SMAD2 and SMAD6/7 inhibit it. SMAD3 deficiency suppresses the expression of type I collagen and prevents the transformation of epithelial cells into myofibroblasts (EMT). In contrast, SMAD2 deficiency upregulates the expression of type I collagen. SMAD4 plays a crucial role in fibrotic diseases by enhancing SMAD3 promoter activity, whereas SMAD6/7 downregulates the fibrotic processes induced by SMAD3. Increasing evidence suggests that the TGFβ/SMAD signaling pathway is involved in organ fibrosis processes, including the liver [20] and kidneys [21]. It is noteworthy that the fibrotic changes mediated by the TGFβ/SMAD pathway are also crucial pathological alterations in elbow stiffness [22, 23], characterized by disrupted extracellular matrix and collagen fiber structure, elevated level of inflammatory cytokines and infiltration of myofibroblasts, ultimately leading to thickening of the elbow joint capsule [24]. These findings align with our results in this study, indicating a significant role of the TGFβ/SMAD signaling pathway in peripheral joint capsule fibrosis. However, conclusive molecular biology evidence is still required to substantiate these findings. Moreover, the genetic correlation among CTS, FS and elbow stiffness is currently under further investigation.

However, this study has certain limitations. Firstly, the subjects of this study are of European ethnicity, so caution should be exercised when applying the results of this study to other ethnicities. Confounding factors such as age, gender, and other environmental factors also have some impact on MR analysis. Nevertheless, this study still provides new insights from a genetic perspective into the genetic causality between CTS and FS, as well as the impact of TGFβ-mediated fibrosis on the pathogenesis of these conditions.

In summary, this study, through GWAS and Mendelian randomization analysis, has preliminarily established carpal tunnel syndrome as a genetic risk factor for frozen shoulder and has also suggested that the fibrosis and myofibroblast transformation mediated by TGFβ/SMAD may play important roles in the pathogenesis of both conditions. The results of this study contribute novel insights to the genetics of frozen shoulder.

### Electronic supplementary material

Below is the link to the electronic supplementary material.

Supplementary Material 1

## Data Availability

The summary-level data of CTS and FS came from the IEU OpenGWAS database (https://gwas.mrcieu.ac.uk/, accessed on September 1^st^, 2023). The MR analysis was performed through the TwoSampleMR packages (version 0.5.6)
