# Supplementary material for: Genetic causal association between frozen shoulder and carpal tunnel syndrome: a two-sample mendelian randomization
Source: BMC Musculoskelet Disord. 2024 Jan 13;25:58. doi: 10.1186/s12891-024-07186-7 (PMC10790250; doi:10.1186/s12891-024-07186-7)
Supplement: Supplementary file 1 — Supplementary Material 1 [file 12891_2024_7186_MOESM1_ESM.docx]

**Table S1 Basic information about GWAS data analysis**

| **GWAS ID** | **Sample size** | **Number of SNPs** |
| --- | --- | --- |
| ukb-d-G6_CARPTU | 361,194 | 12,867,910 |
| ebi-a-GCST90000512 | 451,099 | 15,184,371 |

**Table S2 Forward GWAS data preprocessing and SNP selection**

| **SNP** | **beta.exposure** | **pval.exposure** | **beta.outcome** | **pval.outcome** |
| --- | --- | --- | --- | --- |
| rs12595031 | 0.00188944 | 7.09E-08 | 0.00349176 | 0.79 |
| rs17843768 | -0.00268035 | 1.50E-07 | 0.00723497 | 0.73 |
| rs1866745 | 0.00227484 | 3.47E-10 | 0.0118075 | 0.44 |
| rs3129942 | 0.00231926 | 3.61E-09 | 0.0207613 | 0.19 |
| rs4919190 | 0.00211213 | 1.27E-09 | 0.00330689 | 0.82 |
| rs62167688 | 0.00274612 | 1.35E-10 | 0.0422837 | 0.014 |
| rs62621197 | 0.00535611 | 2.34E-08 | 0.0901952 | 0.019 |
| rs6843953 | 0.00267874 | 8.72E-08 | -0.0255121 | 0.2 |
| rs6977081 | -0.00218673 | 3.38E-09 | -0.0230386 | 0.11 |
| rs72755233 | 0.00365709 | 1.89E-11 | -3.47E-05 | 0.99 |
| rs7649317 | -0.00183445 | 3.19E-07 | -9.06E-05 | 0.99 |
| rs847139 | -0.00232669 | 4.10E-08 | -0.00294542 | 0.88 |

**Table S3 Reverse GWAS data preprocessing and SNP selection**

| **SNP** | **beta.exposure** | **pval.exposure** | **beta.outcome** | **pval.outcome** |
| --- | --- | --- | --- | --- |
| rs1042704 | 0.103074 | 1.10E-09 | 0.000702 | 0.094829 |
| rs117999064 | 0.304439 | 4.20E-08 | 0.002975 | 0.029994 |
| rs12915503 | 0.086278 | 6.40E-08 | -0.00012 | 0.770905 |
| rs13107325 | 0.134162 | 3.50E-07 | 0.000235 | 0.720812 |
| rs2598109 | 0.096864 | 2.70E-07 | -0.00063 | 0.178017 |
| rs28971325 | 0.184128 | 5.30E-29 | -0.00063 | 0.1233 |
| rs530125017 | 0.356925 | 1.40E-07 | -0.00172 | 0.306195 |
| rs6330 | 0.076142 | 5.20E-08 | -1.35E-05 | 0.968865 |
